# Study on the optimal antagonistic effect of a bacterial complex against *Monilinia fructicola* in peach

**DOI:** 10.1515/biol-2020-0080

**Published:** 2020-12-15

**Authors:** Haotian Chang, Haiqing Yang, Tao Han, Fang Wang, Yueping Liu

**Affiliations:** College of Bioscience and Resources Environment, Beijing Advanced Innovation Center for Tree Breeding by Molecular Design, Beijing University of Agriculture, Beijing, 102206, China; Pinggu District of Fruit Bureau, Beijing, 101200, China; Food Science and Engineering College, Beijing University of Agriculture, Beijing, 102206, China

**Keywords:** peach, biocontrol, optimal combination, screening

## Abstract

Peach brown rot caused by *Monilinia fructicola* is one of the most economically destructive diseases of peach (*Prunus persica* L.) in some orchards of China. Biocontrol is a significant strategy that exhibits strong levels of control and ecologically sound concepts in disease management. The purpose of this study was to investigate the combined suppressive effects of three endophytic bacterial strains (xj-14, xj-15, and xj-16) and two soil rhizosphere bacterial strains (xj-A and xj-C) that were shown to have strong inhibitory activity toward *M. fructicola* in our previous study. The optimal strains and the optimized combination of strains were determined. The combination of strains xj-15 and xj-C inhibited *M. fructicola* more intensively for a longer period of time. Following the application of 1 × 10^9^ CFU/mL bacterial complex to the fruits, leaves, and shoots of peach trees infected with *M. fructicola*, the rate of inhibition reached 73.80%, 83.33%, and 90.43%, respectively. A pot experiment using lettuce (*Lactuca sativa*) showed that inoculation with the bacterial complex significantly increased the growth of seedlings. In this study, some compound bacteria were more effective than those in previous study in suppressing disease and promoting growth, which have the potential to be further applied in the field.

## 1 Introduction

Brown rot is a disease caused by the fungus *Monilinia fructicola* (G. Winter) Honey. It can occur during the whole growth period of peach (*Prunus persica* L.) and affect blooms, twigs, and fruit [1]. Brown rot affects the leaves, stems, and fruits of peach trees and other stone fruit trees, including apricot, plum, and cherries [2,3,4,5]. *Monilinia fructicola* lives through the winter as mycelia in overwintered fruit mummies or lesions on peach shoots. It produces a large number of conidia in the following spring, and they are disseminated by wind, rain, and insects [1,6]. The fungus infects the plant through wounds caused by insects, mechanical wounds, or natural cavities in the body of the tree, so the plant is susceptible to *M. fructicola*, particularly under conditions of prolonged rain or high humidity in rainy autumn. Fruits infected with *M. fructicola* rot at high levels, causing enormous economic losses [7].

Currently, chemical control is still one of the most important measures to control brown rot of peach. The main fungicides used include tebuconazole, propiconazole, fluorosilazole, and nitrile oxazole, among others [8]. However, these agents have been used continuously for many years, and pathogens have become resistant to them, resulting in a reduced ability to control pathogens [9]. Biological control using microbes and their metabolites can inhibit or kill plant pathogens without leaving pesticide residues in fruits and it has the additional functions of improving soil and maintaining its ecological balance; this combination is conducive to the sustainable development of agriculture, so the use of biological control for pests and diseases has attracted much attention [10]. Currently, the primary type of biological control used against brown rot of peach is *Bacillus subtilis* (Ehrenberg) Cohn, which is frequently used in the field as a biofungicide [11,12,13].

Despite the sole use of this biofungicide in the biological control of plant diseases, it shows problems of poor adaptability. It has been shown to be ineffective in controlling pathogens in the field, which has resulted in inefficient and inconsistent disease suppression [14]. Therefore, the research and application of complex microbes are urgently needed to increase their utilization in the field. The complementary functions of different combinations of antagonistic bacteria increase their capacity to resist plant diseases. It has been reported that the survival rate of transplanted camphor seedlings treated with complex biofungicides was 30% higher than that of the control group. Moreover, the contents of soluble sugars, chlorophyll, and proline in transplanted camphor seedlings were significantly higher than those of the control group [15]. The effect of complex biofungicides comprising *B. subtilis* and different combinations and proportions of bacterial strains on the control of strawberry anthracnose was much higher than that of a single bacterial agent, and these treatments significantly reduced the incidence of strawberry anthracnose [16]. Additional studies showed that complex biofungicides caused both disease suppression and the promotion of plant growth [17,18]. However, the possibility of competition for space and nutrients among different antifungal agents merits consideration [19]. More research needs to be conducted to explore the optimal combination of different antagonistic bacteria and the effects of the microbial complexes.

Three strains of *B. subtilis* (Ehrenberg) Cohn (xj-14, xj-15, and xj-16), one strain of *B. tequilensis* Gatson (xj-A), and one strain of *B. methylotrophicus* (xj-C) with strong antagonistic activity toward peach brown rot were isolated in our previous research [20,21]. However, each individual strain was effectively antagonistic only for a short time. Therefore, it is necessary to explore the optimal combination of these five antagonistic bacteria to achieve a longer period of disease control. The optimal culture conditions of the bacterial complex were explored. The inhibitory effect of the fermentation broth on tissues of peach trees *in vitro* was studied to obtain a better complex bacterial preparation that is highly effective in controlling brown rot of peach and can lay a foundation for utilization in the field.

## 2 Materials and methods

### 2.1 Source of the antagonistic bacteria and pathogen

Three strains of *B. subtilis* designated xj-14, xj-15, and xj-16 were isolated from the roots of peach trees. One strain of *B. tequilensis* designated xj-A and one strain of *B. methylotrophicus* designated xj-C were isolated from the rhizosphere soil of peach trees. *Monilinia fructicola*, the pathogenic fungus that causes peach brown rot, was isolated from peach orchards in the Pinggu District of Beijing in China and stored at 4–10°C in the Key Laboratory for Northern Urban Agriculture Ministry of Agriculture and Rural Affairs.

### 2.2 Antagonistic activity among bacteria

The Oxford cup method was used to conduct the experiment on the exclusionary effects among bacteria [22]. The fermentation broth of one strain was poured into a culture dish. After the media had cooled and solidified, tweezers were used to place a sterile Oxford cup on the medium. Next, the fermentation broth of other strains was poured into the Oxford cup. The amount of strains in the Oxford cup was 0.5, 1.0, 1.5, and 2.0 times greater than that mixed in the plate. After 2 days of incubation, there was no obvious antagonism between the strains mixed in the plate and the strains poured in the Oxford cup if there was no inhibitory zone around the Oxford cup. In contrast, there was antagonism between the two strains if an obvious inhibitory zone appeared. In this experiment, 30 combinations were examined, and the most promising combination was selected for further study. All the treatments were repeated in triplicate.

### 2.3 Detection of the antagonistic effect of bacteria on *M. fructicola*

The inhibitory effect of the fermentation broth on brown rot of peach was determined using the filter paper method [23]. A block of the pathogenic fungus on agar was placed in the center of a Petri dish that contained Potato Dextrose Agar medium, and six pieces of aseptic filter paper were placed at a distance of 1 cm from the edge of the dish. The same volume of fermentation broth of the single strain or the complex mixture of strains was added to the filter paper at ratios of 1:1, 2:1, and 1:2 to detect the antimicrobial effect. The control group was the uninoculated liquid culture medium. Each treatment was repeated in triplicate. The diameter of the pathogenic colony was measured, and the inhibition rate was calculated. The formula for the calculation of the inhibition rate (%) is as follows: (colony diameter of the control group – colony diameter of the treatment group)/(colony diameter of the control group – the diameter of the original block) × 100%.

### 2.4 Optimization of the fermentation conditions of complex bacteria

The optimal media for the complex bacteria were studied using the basic medium as a starting point. An organic nitrogen source (beef extract, yeast extract, peptone, and tryptone) and an inorganic nitrogen source (ammonium sulfate and urea) were used to replace the nitrogen source in the basic medium [24]. The medium without the addition of additional nitrogen was used as the control. Glucose, sucrose, fructose, and soluble starch were used individually to replace the carbon source in the basic medium, and the medium without carbon was used as the control. Each treatment was repeated in triplicate. The bacteria were incubated at 37°C while shaking at 150 rpm for 24 h. Additional conditions of the fermentation culture were optimized based on the optimal nitrogen and carbon source. The temperatures examined were 25, 30, and 35°C. The optimal volumes for inoculation were 1, 3, 5, 7, and 9% of the culture volume. The volume of liquid in each 250 mL flask was 40, 60, 80, 100, and 120 mL. The initial pH values were 5, 5.5, 6, 6.5 and 7, respectively. Each treatment was repeated in triplicate. The antagonistic activity of the fermentation broth was determined as described in Section 2.3.

### 2.5 *In vitro* direct antagonistic effects of the bacterial complex on *M. fructicola*

The antagonistic effect of the bacterial complex on *M. fructicola* was determined as described in ref. [21]. The fermentation broth of complex bacteria was studied using three gradients, i.e., 1 × 10^7^, 1 × 10^8^, and 1 × 10^9^ CFU/mL.

Fresh and disease-free fruits were washed with sterile water to remove dust from their surface. The following process was conducted in a laminar flow hood. The fruits were disinfected in a solution of 1% NaClO for 2 min and rinsed three times with sterile water. The surface was disinfected with 75% alcohol on a sterilized bench and rinsed with sterile water an additional three times before inoculation. A sterile hole punch was used to make a hole approximately 5 mm deep and wide on the equator of each fruit. The fruits with wounds were placed in the fermentation broth for 15 min and then transferred to a new sterile culture dish. A block of *M. fructicola* was introduced into the wounds and kept at 28°C for 3 days followed by observation of the extent of infection.

The surfaces of the leaves and shoots were disinfected by immersion in a solution of 1% NaClO for 2 min and then rinsed three times with sterile water. Leaves at similar developmental stages were wounded at six spots along the main vein at the mesophyll using a sterile scalpel. The wounds were approximately 1 mm in length. Each healthy shoot was wounded once on the phloem using a sterile scalpel. The wounded leaves and shoots were immersed in fermentation broth for 4 h. A block of *M. fructicola* was then placed in each wound in the leaves and shoots before incubation in a 28°C controlled-environment chamber for 3 days followed by observation of the infection extent.

The infections of the fruit, leaves, and shoots by *M. fructicola* were quantified as follows:

\text{Incidence}\hspace{.8em}( \% )=\text{lesion}\hspace{.5em}\text{number}\hspace{.5em}\text{of}\hspace{.5em}\text{treated}\hspace{.5em}\text{fruits/number}\hspace{.5em}\text{of}\hspace{1em}\hspace{.5em}\text{treated}\hspace{.5em}\text{fruits}\times 100 \%

\text{Inhibition}\hspace{.5em}\text{rate}\hspace{.5em}( \% )=\left[\text{lesion}\hspace{.5em}\text{size}\hspace{.5em}\text{of}\hspace{.5em}\text{untreated}\hspace{.5em}\text{fruits}\hspace{.5em}({\text{cm}}^{2})-\text{lesion}\hspace{.5em}\text{size}\hspace{.5em}\text{of}\hspace{.5em}\text{treated}\hspace{.5em}\text{fruits}\hspace{.5em}{\text{(cm}}^{2}\text{)}\right]/\text{lesion}\hspace{.5em}\text{size}\hspace{.5em}\text{of}\hspace{.5em}\text{control}\hspace{.5em}({\text{cm}}^{2})\times 100 \%

### 2.6 Effect of the bacterial complex on the growth of lettuce seeds and seedlings

There were the following eight treatments in this experiment: (I) sterilized water, (II) the culture medium of the bacterial complex, (III) fermentation broth of the bacterial complex, (IV) a 50-fold dilution of the fermentation broth containing the bacterial complex, (V) a 100-fold dilution of the fermentation broth of the bacterial complex, (VI) the autoclaved fermentation broth of the bacterial complex, (VII) a 50-fold dilution of the autoclaved fermentation broth of the bacterial complex, and (VIII) a 100-fold dilution of the autoclaved fermentation broth of the bacterial complex.

Lettuce (*Lactuca sativa*) seeds of the variety “beisansheng no. 2” were incubated for 3 days at 4°C to accelerate germination, and the treated seeds were placed on sterile moistened filter paper in a glass culture dish. After 1 day of culture, the seeds began to germinate. When the embryos appeared, ten seeds of the same size were chosen and placed in glass culture dishes that contained wet filter paper. A total volume of 25 mL of the optimal fermentation broth of the bacterial complex was added to each culture dish. Ten seeds per treatment were used to assess the germination. Ultra-pure water purified using a Millipore system (ELGA Purelab Classic, England) was used to keep the filter paper moist during the culture process. After 5 days, the increase in radicle length, germ length, dry weight, and fresh weight of the seeds was measured.

The germinated lettuce seeds were planted in a full tray of 1:1 peat and vermiculite (v/v). After 7–10 days of culture, the seedlings that had grown were transplanted to a pot (18 cm diameter × 18 cm depth) full of 1:1 peat and vermiculite for growth after the plants had grown four leaves. After 15 days of incubation, lettuce plants with the same growth state were selected for treatment. A total volume of 30 mL of the different treatments of the fermentation broth of the bacterial complex was used to irrigate each pot. The pots were randomly arranged with six replicates per treatment. The plants were grown in a sunlit greenhouse with 25/20°C day/night temperatures and 40–60% relative humidity. After 4 weeks of growth, the root length, plant height, leaf number, dry weight, wet weight, and chlorophyll content of lettuce leaves were measured.

### 2.7 Statistical analysis

The collected data were subjected to one-way analysis of variance using SPSS 20.0 software. The comparison of mean effects was based on Duncan’s new multiple range test at a significance level of 0.05.

## 3 Results

### 3.1 The bacterial complex with the optimal antagonistic effect against *M. fructicola* comprised a combination selected out of five strains

**Figure 1:**
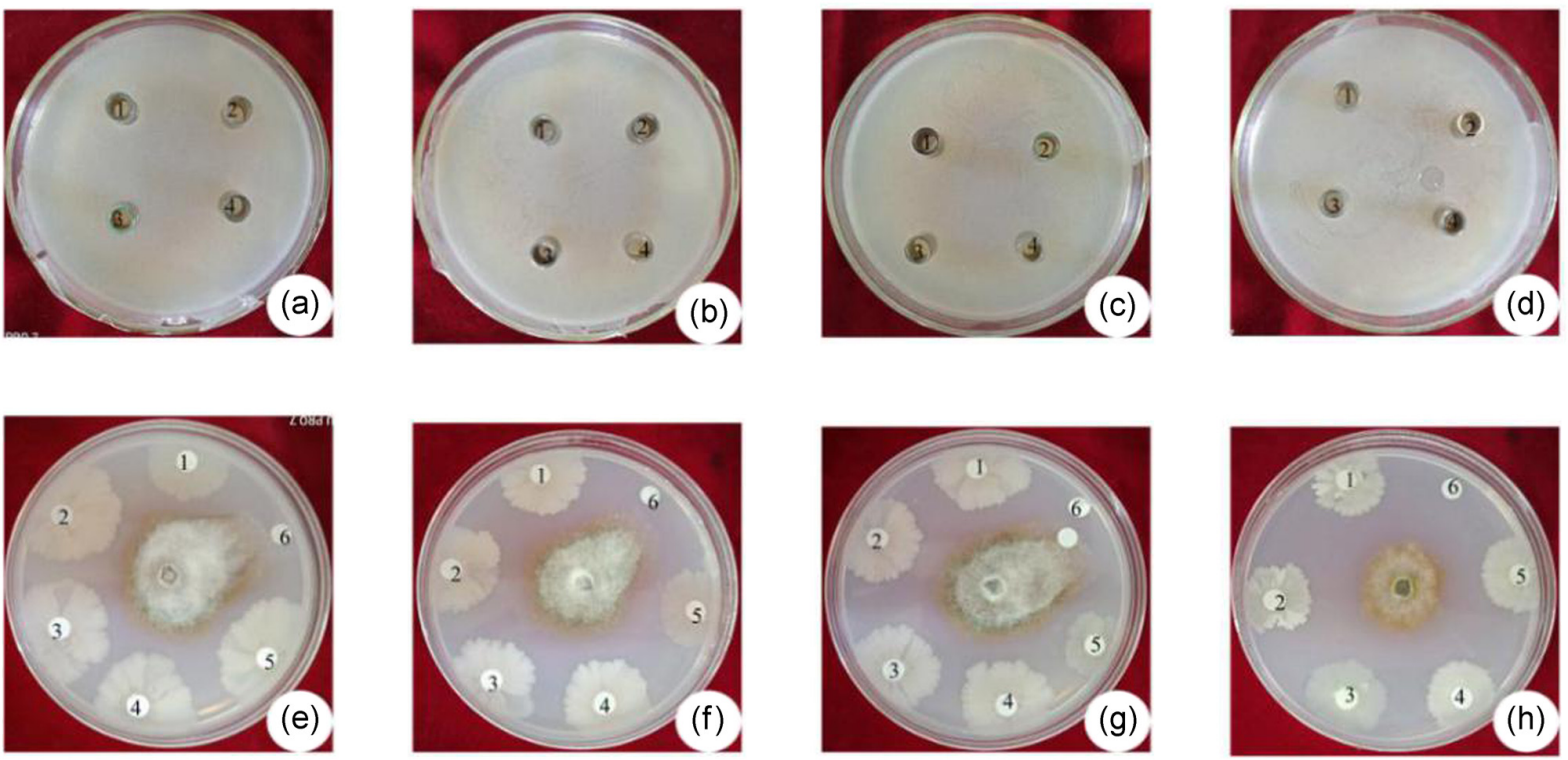
Exclusionary effect among strains at different concentrations and antagonistic effect of different ratios of the bacterial complex on *Monilinia fructicola*. (a–d) The exclusionary effect of strain xj-15 in the medium and the other strain in the Oxford cup with different ratios. The numbers 1–4 in the plate represent the ratio of the two strains (0.5:1, 1:1, 1.5:1, and 2:1, respectively). (e–h) The antagonistic effect of the bacterial complex on *M. fructicola*. The numbers 1–6 in the plate represent a single strain or different combinations of the five strains: E1: xj-14; E2: xj-14:xj-15 = 1:1; E3: xj-14:xj-15 = 2:1; E4: xj-14:xj-15 = 1:2; E5: xj-15; E6: the culture medium. F1: xj-15; F2: xj-15:xj-16 = 1:1; F3: xj-15:xj-16 = 2:1; F4: xj-15:xj-16 = 1:2; F5: xj-16; F6: the culture medium. G1: xj-15; G2: xj-15:xj-A = 1:1; G3: xj-15:xj-A = 2:1; G4: xj-15:xj-A = 1:2; G5: xj-A; G6: the culture medium. H1: xj-15; H2: xj-15:xj-C = 1:1; H3: xj-15:xj-C = 2:1; H4: xj-15:xj-C = 1:2; H5: xj-C; H6: the culture medium.

**Figure 2:**
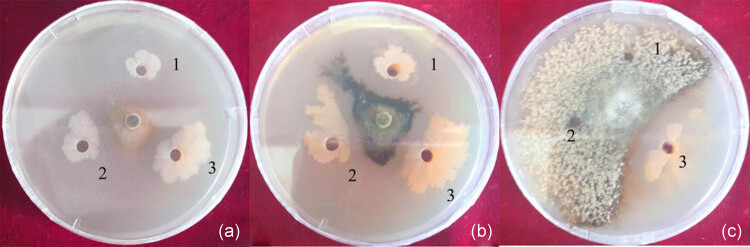
The consistency of the antagonistic effect of the bacterial complex on *Monilinia fructicola.* (a) The antagonistic effect of a single strain and the bacterial complex cultured for 3 days on *M. fructicola*. (b) The antagonistic effect of a single strain and the bacterial complex cultured for 15 days on *M. fructicola*. (c) The antagonistic effect of a single strain and the bacterial complex cultured for 33 days on *M. fructicola*. The numbers 1–3 in the plate represent xj-C, xj-15, and the bacterial complex, respectively.

The degree of antagonism of each type of antagonistic bacterial strain isolated from the soil or endophytes of peach trees may differ when they are mixed with different strains or utilized at different concentrations. An examination of 30 combinations and four different concentration gradients indicated that the antagonistic effect among the five strains, including xj-14, xj-15, xj-16, xj-A, and xj-C, was present only in xj-16 and xj-C when a ratio of 1:2 was applied. No antagonistic effect was found among the other four strains, which indicated that any combination of these strains has the potential to serve as a complex of antimicrobial agents (Figure 1a–d). The combination of xj-15 and xj-C had the strongest antagonistic ability against *M. fructicola* with a rate of antagonism that was as high as 54%, 15%, and 2% greater than that of the single strains xj-15 and xj-C (Table 1). The pathogenic mycelia that were inhibited had a small range of distribution (Figure 1e and f). In the initial stage of culture, the antagonistic effects of the combined bacterial strains xj-15 and xj-C on *M. fructicola* were not observable (Figure 2a and b). However, after 33 days of culture, the combined bacterial mixture still had a strongly antagonistic effect toward *M. fructicola*, while the single xj-15 and xj-C strains completely lost their antagonistic ability (Figure 2c).

**Table 1:** Antagonistic effect of five single strains and the bacterial complex on the growth of *Monilinia fructicola*

Culture mode	Plate number	Combination ratio	Strains	Antagonistic radius (mm)	Inhibition rate (%)
Single strain culture	E6, F6, G6, H6		Culture medium	23.226	0
	E1		xj-14	9.05 ± 0.15	39.12
	E5, F1, G1, H1		xj-15	9.14 ± 0.15	39.23
	F5		xj-16	6.90 ± 0.15	30.11
	G5		xj-A	10.47 ± 0.10	45.12
	H5		xj-C	12.05 ± 0.22	52.21
Different ratios of bacteria in the complex	E2	1:1	xj-14:xj-15	10.64 ± 0.11b	46.12
	E3	2:1	xj-14:xj-15	9.82 ± 0.09b	42.45
	E4	1:2	xj-14:xj-15	9.79 ± 0.07cd	42.14
	F2	1:1	xj-15:xj-16	9.39 ± 0.171b	40.18
	F3	2:1	xj-15:xj-16	8.48 ± 0.255c	36.54
	F4	1:2	xj-15:xj-16	9.11 ± 0.417c	39.23
	G2	1:1	xj-15:xj-A	8.59 ± 0.245d	37.18
	G3	2:1	xj-15:xj-A	8.34 ± 0.318d	36.51
	G4	1:2	xj-15:xj-A	9.97 ± 0.265b	43.62
	H2	1:1	xj-15:xj-C	12.54 ± 0.167a	54.92
	H3	2:1	xj-15:xj-C	12.37 ± 0.102a	53.18
	H4	1:2	xj-15:xj-C	12.43 ± 0.159a	54.12

Note: “±” represents the standard deviation, whereas lowercase letters represent a significant difference (Duncan’s test, *P* < 0.05).

### 3.2 Optimization of the culture conditions of the bacterial complex

#### 3.2.1 Optimization of carbon and nitrogen sources for the bacterial complex

Table 2 shows that the highest antagonistic radius of the fermentation broth of 13.2 and 11.1 mm was reached when glucose was used as the carbon source and yeast extract was used as the nitrogen source, respectively. Therefore, glucose and yeast extract were selected as the carbon and nitrogen sources for the bacterial complex.

**Table 2:** Antagonistic effect of the fermentation broth of the bacterial complex with different carbon and nitrogen sources against *Monilinia fructicola*

Nitrogen source	Antagonistic radius (mm)	Carbon source	Antagonistic radius (mm)
Tryptone	8.26 ± 1.27c	Glucose	13.2 ± 0.97a
Peptone	8.01 ± 0.69c	Sucrose	12.2 ± 1.2ab
Yeast extract	11.10 ± 0.67a	Soluble starch	9.90 ± 1.64ab
Beef extract	10.14 ± 0.72b	Fructose	11.4 ± 1.68b
Ammonium sulfate	6.60 ± 0.91d		
Urea	10.73 ± 0.83ab		

Note: “±” represents the standard deviation, and lowercase letters represent a significant difference (Duncan’s test, *P* < 0.05).

#### 3.2.2 Optimization of the fermentation conditions of the bacterial complex

**Figure 3:**
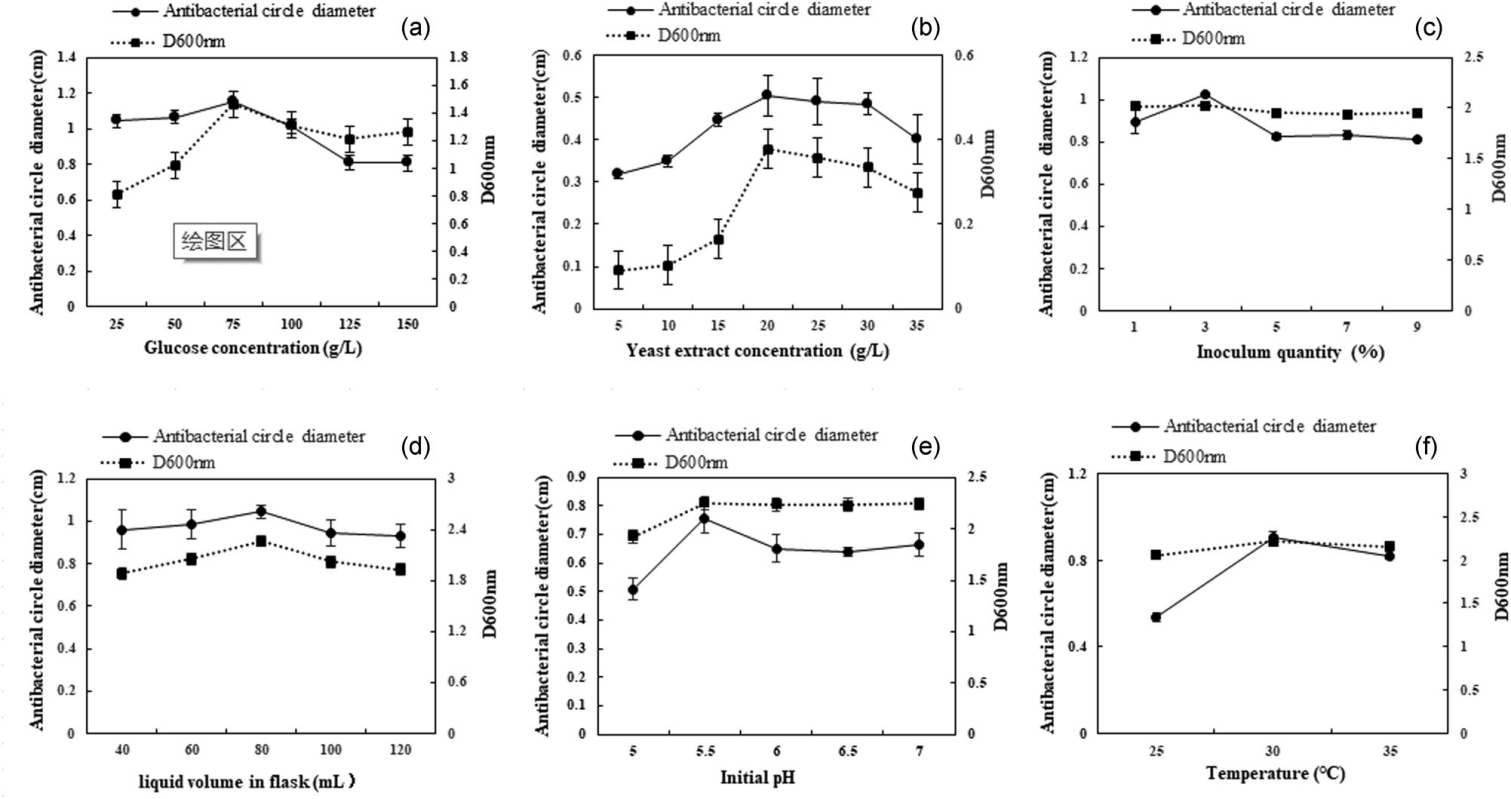
Optimization of the fermentation conditions of the bacterial complex against *Monilinia fructicola*. (a) The concentration of glucose. (b) The concentration of yeast extract. (c) The quantity of inoculum. (d) The volume of liquid in flask. (e) The initial pH. (f) The temperature of the fermentation.

To optimize the fermentation conditions of the bacterial complex selected, the optimal conditions were explored through the evaluation of the antibacterial circle diameter and the concentration of bacteria. The optimal concentrations of glucose and yeast extract were 75 and 20 g/L, respectively (Figure 3a and b). The quantity of inoculum was 3% (Figure 3c). The ratio of liquid volume to the flask volume was 100:250 (Figure 3d). The suitable pH was 5.5, and the optimal temperature for the growth of bacteria was 30°C (Figure 3e and f).

#### 3.2.3 The fermentation broth of the bacterial complex cultured with the optimized medium increases the antagonistic ability against *M. fructicola*

The rate of inhibition of the bacterial complex against *M. fructicola* was 46% and 50%, respectively, when the fermentation broth was cultured under the basic conditions. The rate of inhibition reached 71% when the conditions of fermentation were optimized (Table 3). The optimized culture conditions increased the antagonistic activity of the bacterial complex, and they were adopted in the following experiment.

**Table 3:** Antagonistic effect of the fermentation broth of the bacterial complex under different culture conditions against *Monilinia fructicola*

Different culture conditions	Antagonistic radius (mm)	Inhibition rate (%)
Medium for a single strain	11.05 ± 0.10b	46.12
Basic medium	12.15 ± 1.41b	50.34
Optimized medium	18.15 ± 0.44a	71.09

Note: “±” represents the standard deviation, whereas lowercase letters represent a significant difference (Duncan’s test, *P* < 0.05).

### 3.3 Antagonistic ability of bacteria against *M. fructicola* on peach plants *in vitro*

**Figure 4:**
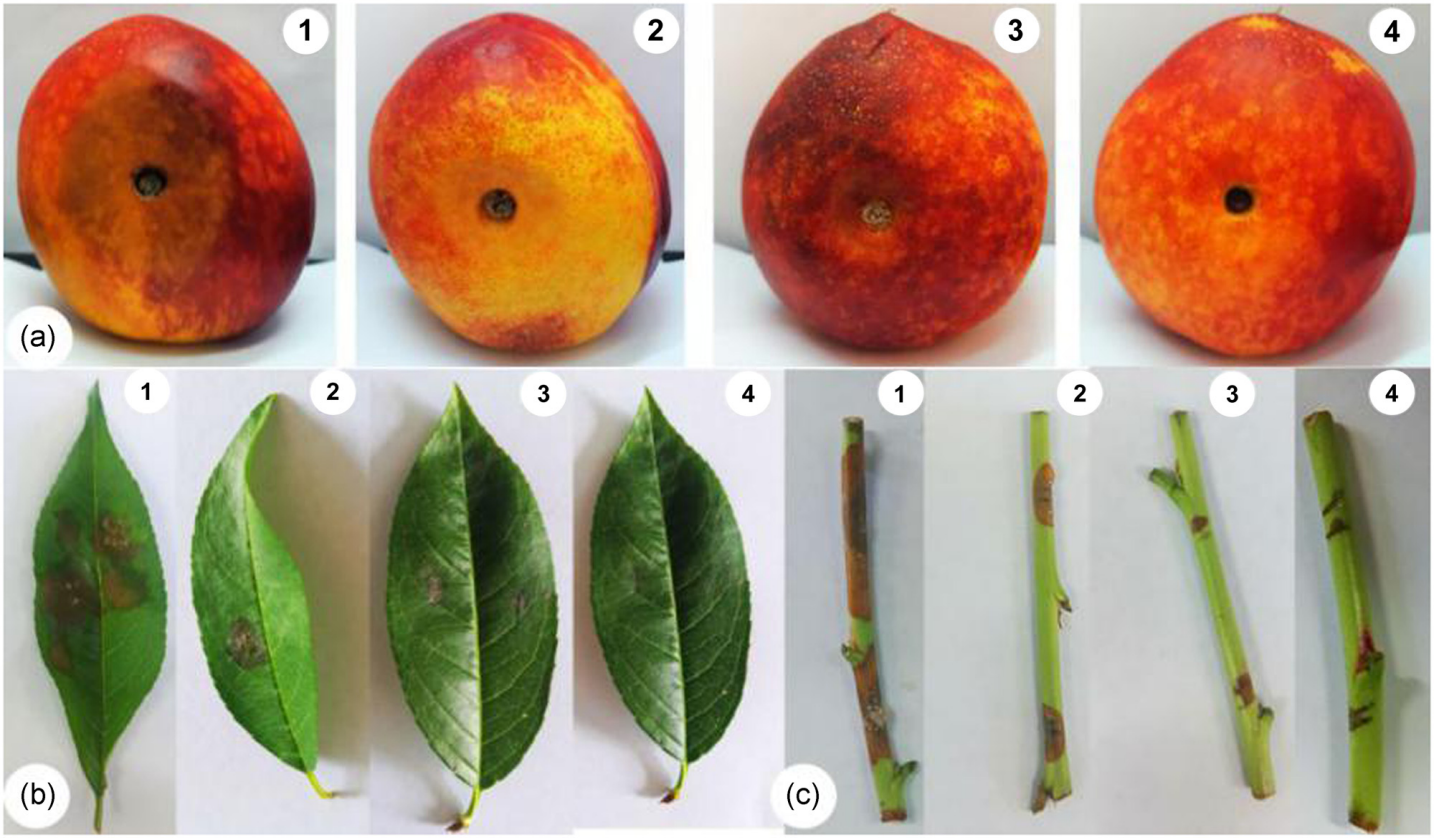
The antagonistic ability of the bacterial complex against *Monilinia fructicola* in the fruits, leaves, and shoots of a peach tree. (a) Peach fruit. (b) Peach leaves. (c) Peach branches. (1) Water treatment as the control; (2) bacterial complex fermentation broth with a concentration of 1 × 10^7^ CFU/mL; (3) bacterial complex fermentation broth with a concentration of 1 × 10^8^ CFU/mL; and (4) bacterial complex fermentation broth with a concentration of 1 × 10^9^ CFU/mL.

Peach fruit, leaves, and branches were treated *in vitro* with the fermentation broth from the bacterial complex at 1 × 10^7^, 1 × 10^8^, and 1 × 10^9^ CFU/mL. The fermentation broth at different concentrations was antagonistic against *M. fructicola* (Figure 4). The antagonistic effect became much stronger as the concentration of the fermentation broth increased. At a fermentation broth concentration of 1 × 10^9^ CFU/mL, the rate of inhibition reached values as high as 73.80%, 83.33%, and 90.40% for peach fruit, leaves, and branches, respectively; moreover, the incidence was low (Table 4).

**Table 4:** Antagonistic effect of the bacterial complex against *Monilinia fructicola* in fruits, leaves, and shoots treated with different concentrations of fermentation broth

Different peach tree tissues	Different concentrations of fermentation broth (CFU/mL)	Incidence (%)	Lesion diameter (cm)	Rate of inhibition (%)
Fruits	a: control	100.00a	9.24 ± 0.30d	—
	b: 1 × 10^7^	100.00a	7.62 ± 0.33d	17.53
	c: 1 × 10^8^	66.71b	5.20 ± 0.28d	43.72
	d: 1 × 10^9^	33.33c	2.42 ± 0.78c	73.80
Leaves	a: control	100.00a	0.72 ± 0.31a	—
	b: 1 × 10^7^	81.57b	0.38 ± 0.17a	47.22
	c: 1 × 10^8^	73.78b	0.27 ± 0.10a	60.01
	d: 1 × 10^9^	42.45c	0.12 ± 0.05b	83.33
Branches	a: control	100.00a	2.50 ± 0.31c	—
	b: 1 × 10^7^	66.67b	1.30 ± 0.31b	48.13
	c: 1 × 10^8^	33.33c	0.62 ± 0.08a	75.21
	d: 1 × 10^9^	33.33c	0.24 ± 0.03a	90.43

Note: “±” represents the standard deviation, whereas lowercase letters represent a significant difference (Duncan’s test, *P* < 0.05).

### 3.4 Effect of the bacterial complex on the germination of lettuce seeds and growth of seedlings

**Figure 5:**
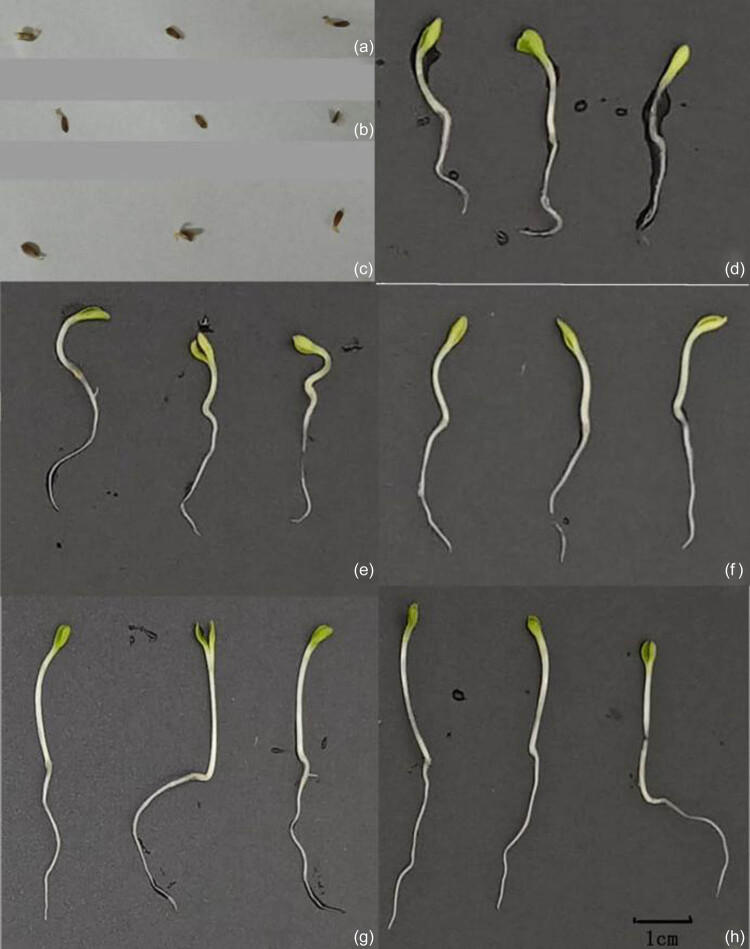
Effect of different treatments of the fermentation broth of the bacterial complex on germination of lettuce seeds and growth of seedlings. (a) Fermentation broth of the bacterial complex (I). (b) The autoclaved fermentation broth of the bacterial complex (II). (c) The culture of the bacterial complex (III). (d) Sterilized water (IV). (e) 50-fold dilution of the fermentation broth of the bacterial complex (V). (f) 100-fold dilution of the fermentation broth of the bacterial complex (VI). (g) 50-fold dilution of the autoclaved fermentation broth of the bacterial complex (VII). (h) 100-fold dilution of the autoclaved fermentation broth of the bacterial complex (VIII).

Lettuce seeds could not germinate in the following culture media used for the growth of the bacterial complex: the culture medium, fermentation broth, and autoclaved fermentation broth (Figure 5a–c). The lettuce seeds germinated well in the diluted and autoclaved diluted fermentation broths. The increase in radicle length and fresh weight was much higher in seeds treated with the control and autoclaved fermentation broths that had been diluted 100-fold compared to that of the other treatments (Table 5).

**Table 5:** Effect of different treatments of the fermentation broth of the bacterial complex on the germination and growth of lettuce seeds

Treatment	Radicle length (mm)	Dry weight (mg)	Fresh weight increase (mg)
I	0	0	0
II	0	0	0
III	0	0	0
IV	13.18 ± 0.67b	3.76 ± 0.75b	35.40 ± 1.49b
V	4.534 ± 0.73c	6.08 ± 1.22a	34.11 ± 2.00b
VI	23.94 ± 0.39a	6.32 ± 1.26a	51.94 ± 1.44a
VII	15.96 ± 0.52b	4.56 ± 0.42b	29.29 ± 1.09b
VIII	22.10 ± 0.36a	4.66 ± 0.93b	51.43 ± 1.18a

Note: “±” represents the standard deviation, whereas lowercase letters represent a significant difference (Duncan’s test, *P* < 0.05).

(I) Fermentation broth of the bacterial complex; (II) the autoclaved fermentation broth of the bacterial complex; (III) the culture of the bacterial complex; (IV) sterilized water; (V) 50-fold dilution of the fermentation broth of the bacterial complex; (VI) 100-fold dilution of the fermentation broth of the bacterial complex; (VII) 50-fold dilution of the autoclaved fermentation broth of the bacterial complex; (VIII) 100-fold dilution of the autoclaved fermentation broth of the bacterial complex.

The strongest promotion of growth was observed following treatment with the control or autoclaved fermentation broth diluted 100-fold. The root length, plant height, and fresh weight of lettuce plants treated with fermentation broth diluted 100-fold (treatment VI) increased by 1.2, 3.1, and 7.5 times, respectively, compared with the treatment with the culture medium (treatment IV; Table 6). The leaf number and the chlorophyll content of treatment VI were the highest among all treatments. No difference in the content of vitamin C was observed between treatments IV and VI.

**Table 6:** Effect of different fermentation broth treatments of the bacterial complex on the growth of lettuce seedlings

Treatment	Root length (cm)	Plant height (cm)	Fresh weight (g)	Number of leaves	Chlorophyll content (mg/g)	Vitamin C content (mg/g)
I	8.93 ± 0.21a	9.87 ± 0.19bc	2.94 ± 0.19c	11 ± 2d	11.30 ± 0.67a	6.61 ± 1.87b
II	9.30 ± 0.51a	10.13 ± 0.43bc	4.54 ± 0.43d	11 ± 2d	13.3 ± 2.05ab	7.66 ± 0.22bc
III	8.86 ± 1.03a	8.21 ± 0.54b	1.17 ± 0.23a	7 ± 1ab	12.03 ± 2.95a	7.66 ± 0.1bc
IV	14.2 ± 0.51bc	4.47 ± 3.04a	1.22 ± 0.18ab	5 ± 1a	15.3 ± 3.26ab	8.91 ± 0.42c
V	13.5 ± 1.75b	12.03 ± 0.65cd	2.33 ± 0.27c	6 ± 1ab	13.03 ± 1.17ab	5.91 ± 1.85ab
Ⅵ	16.43 ± 0.95c	13.77 ± 1.30d	9.14 ± 0.89e	14 ± 1e	17.93 ± 1.99c	9.94 ± 0.58c
VII	11.73 ± 0.82ab	10.5 ± 0.78bc	2.09 ± 0.32bc	9 ± 2bc	13.27 ± 3.48ab	5.67 ± 0.17ab
VIII	10.43 ± 2.68a	12.77 ± 0.61cd	2.42 ± 0.30c	8 ± 1bc	10.53 ± 2.41a	3.68 ± 0.72a

Note: “±” represents the standard deviation, whereas lowercase letters represent a significant difference (Duncan’s test, *P* < 0.05).

(I) Fermentation broth of the bacterial complex; (II) the autoclaved fermentation broth of the bacterial complex; (III) the culture of the bacterial complex; (IV) sterilized water; (V) 50-fold dilution of the fermentation broth of the bacterial complex; (VI) 100-fold dilution of the fermentation broth of the bacterial complex; (VII) 50-fold dilution of the autoclaved fermentation broth of the bacterial complex; (VIII) 100-fold dilution of the autoclaved fermentation broth of the bacterial complex.

## 4 Discussion

Five strains of *Bacillus* xj-14, xj-15, xj-16, xj-A, and xj-C that were antagonistic toward *M. fructicola* were utilized in this experiment. A total of 30 experimental combinations were designed at three levels (1:1, 2:1, and 1:2). There was almost no strong exclusionary reaction between any of the two combinations of the five strains. However, when the ratio of strains xj-16 and xj-C was 1 to 2, an obvious inhibitory circle appeared. These results have been reported previously [20]. When multiple strains were cultured together, there was a certain antagonistic effect, which required adjustment. The ratio of strains and order of inoculation were used to reduce the antagonism of each strain in co-culture. Our results showed that the mixed culture of different strains was not suitable for all applications.

The plate confrontation method proved that the combination of strains xj-15 and xj-C was the most active against *M. fructicola* at all levels. The rate of antagonism reached 54%, which was higher than that of any of the five strains separately (Figure 1). Moreover, this value was determined when the combination of strains xj-15 and xj-C (Figure 2) was cultured for longer duration, which indicated that the combination of the two strains had a strong synergistic effect. Strain xj-C is an isolate of *B. methylotrophicus*. There are few reports about its application in biological control compared with other *B. subtilis*, and even fewer reports about its application combined with other strains, particularly for the control of *M. fructicola*. However, the application of a powdered preparation of *B. methylotrophicus* WF-3 was more effective than other biofungicides in protecting against cucumber anthracnose [25]; the antagonistic effect was as high as 77.38% and 72.69% compared with the control, respectively. The effect of strain xj-15 on the biological control of *M. fructicola* was stronger in the previous study [20]. In this experiment, although the single strains xj-14 and xj-16 demonstrated the strongest antagonistic ability, the rate of inhibition of the combination did not exceed that of the single strain xj-16 in any ratio of the two strains. These results showed that the effect produced by the combination of strains was quite different; it was lower or higher than that of a single strain, or no significant effect was observed [26]. The bacterial complex requires optimization by more experiments.

The conditions of fermentation affected the field application of complex bacteria [27]. In this study, the most suitable carbon and nitrogen sources for the production of the bacterial complex were optimized using a single factor test. The results showed that with different carbon and nitrogen sources, the growth of strains positively correlated with the antimicrobial activity to some extent. Glucose as the carbon source and yeast extract as the nitrogen source aided the growth of the bacterial complex. The other optimal fermentation conditions, including the quantity of inoculum, the liquid volume in flask, the initial pH, and the temperature of the fermentation, were obtained. The antimicrobial activity of the bacterial complex increased under the optimized fermentation conditions.

On the basis of the optimal bacterial complex obtained, the effects of this complex against *M. fructicola* in peach plants *in vitro* and on the growth of lettuce seeds and seedlings were studied. To explore the occurrence of *M. fructicola in vitro*, three healthy types of peach tissues, including fruits, branches, and leaves, were selected for evaluation using three different concentrations of the fermentation broth. The results showed that the fermentation broth of the optimal bacterial complex decreased the incidence of tissue infection and increased the rate of inhibition, which showed that the bacterial complex had a substantial antagonistic effect on *M. fructicola*. However, more research studies need to be developed to verify the antagonistic effect of the bacterial complex in the presence of complex soil microorganisms and under environmental conditions that the bacteria would encounter during application in the field. Additionally, the bacterial complex had a notable effect on the germination of lettuce seeds and seedling growth. The bacterial complex comprising rhizosphere soil isolates of strain xj-C can prevent infections by some plant pathogens [25,28,29]. Moreover, the rhizosphere soil bacteria can promote plant growth [30,31]. The main reason was the production of plant growth-promoting substances synthesized by bacteria or the promotion of the absorption of nutrients in the growing environment [32,33]. Through our study, we obtained a complex biofungicide with strong resistance to *M. fructicola* and growth promoting activity, which can be further applied in the field following additional research.

## References

[1] Zhu XQ, Chen XY, Luo Y, Guo LY. First report of Monilinia fructicola on peach and nectarine in China. Plant Pathol. 2005;54(4):575.

[2] Boehm EW, Ma Z, Michailides TJ. Species-specific detection of Monilinia fructicola from California stone fruits and flowers. Phytopathology. 2001;91(5):428–39. 10.1094/PHYTO.2001.91.5.428 18943587

[3] Li SF, Chen C. Incidence and management of the peach fruit brown rot. Plant Prot. 2009;6(8):3373–86.

[4] Ma ZH, Michael AY, Themis JM. Identification and characterization of benzimidazole resistance in Monilinia fructicola from stone fruit orchards in California. Appl Environ Microbiol. 2003;69(12):7145–52. 10.1128/AEM.69.12.7145-7152.2003 PMC309941 14660360

[5] Oro L, Feliziani E, Ciani M, Romanazzi G, Comitini F. Biocontrol of postharvest brown rot of sweet cherries by Saccharomyces cerevisiae Disva 599, Metschnikowia pulcherrima Disva 267 and Wickerhamomyces anomalus Disva 2 strains. Postharvest Biol Technol. 2014;96:64–8.

[6] Emery KM, Michailides TJ, Scherm H. Incidence of latent infection of immature peach fruit by Monilinia fructicola and relationship to brown rot in Georgia. Plant Dis. 2000;84(8):853–7. 10.1094/PDIS.2000.84.8.853 30832138

[7] Ritchie D. Mycelial growth, peach fruit rotting capability, and sporulation of strains of Monilinia fructicola resistant to Dichloran, Iprodione, Procymidone, and Vinclozolin. Phytopathology. 1983;73:44–7.

[8] Yang LY, Zhang JL, Bassett CL, Meng XH. Difference between chitosan and oligochitosan in growth of Monilinia fructicola and control of brown rot in peach fruit. LWT – Food Sci Technol. 2012;46:254–9.

[9] Zhang Y, Zeng L, Yang J, Zheng X, Yu T. 6-Benzylaminopurine inhibits growth of Monilinia fructicola and induces defense-related mechanism in peach fruit. Food Chem. 2015;187:210–7. 10.1016/j.foodchem.2015.04.100 25977018

[10] Dukare AS, Paul S, Nambi VE, Gupta RK, Singh R, Sharma K. Exploitation of microbial antagonists for the control of postharvest diseases of fruits: a review. Crit Rev Food Sci Nutr. 2018;59(9):1498–513. 10.1080/10408398.2017.1417235 29336595

[11] Liu C, Yin X, Wang Q, Peng Y, Ma Y, Liu P, et al. Antagonistic activities of volatiles produced by two bacillus strains against Monilinia fructicola in peach fruit. J Sci Food Agric. 2018;98(15):5756–63. 10.1002/jsfa.9125 29756313

[12] Wang Z, Wang Y, Zheng L, Yang X, Liu H, Guo J. Isolation and characterization of an antifungal protein from Bacillus licheniformis hs10. Biochem Biophys Res Commun. 2014;454(1):48–52. 10.1016/j.bbrc.2014.10.031 25445597

[13] Wang NN, Yan X, Gao XN, Niu HJ, Kang ZS, Huang LL. Purification and characterization of a potential antifungal protein from Bacillus subtilis E1R-J against Valsa mali. World J Microbiol Biotechnol. 2016;32(4):63. 10.1007/s11274-016-2024-5 26925625

[14] Eljounaidia K, Lee SK, Baea H. Bacterial endophytes as potential biocontrol agents of vascular wilt diseases – review and future prospects. Biol Control. 2016;103:62–8.

[15] Ding LP, Sun WH, Li PF. Effect of composite microbial agents on transplant of Cinnamomum camphora seedings and nursery soil. Curr Biotechnol. 2017;3:236–40.

[16] Chen Z, Huang J, Zhao J, Liang H. Screening of the combinations of Bacillus strains against strawberry Anthracnose. Chin J Biol Control. 2018;34(4):582–8.

[17] Li QF, Deng X, Wu CY, Cao EH. Effects of complex bacterium on growth and disease resistance of tomato. Ecol Environ Sci. 2012;21(11):1836–40.

[18] Tamayo-Velez A, Osorio NW. Co-inoculation with an arbuscular mycorrhizal fungus and a phosphate-solubilizing fungus promotes the plant growth and phosphate uptake of avocado plantlets in a nursery. Botany. 2017;95(5):539–45.

[19] Hu JL, Hou SW, Li MH, Wang JH, Wu FY, Lin XG. The better suppression of pepper Phytophthora blight by arbuscular mycorrhizal (AM) fungus than Purpureocillium lilacinum alone or combined with AM fungus. J Soils Sediment. 2020;20:792–800.

[20] Hou X, Zhang GQ, Hu X, Liu YP. Screening and inhibitory effect of antagonistic endophytic bacteria in peach against Monilinia fructicola. Microbiol China. 2017;44(8):1874–81.

[21] Yuan X, Hou X, Chang H, Yang R, Wang F, Liu YP. Bacillus methylotrophicus has potential applications against Monilinia fructicola. Open Life Sci. 2019;14:410–9. 10.1515/biol-2019-0046 PMC7874823 33817176

[22] Wang Y, Lu ZX, Wu H, Lv FX. Study on the antibiotic activity of microcapsule curcumin against foodborne pathogens. Int J Food Microbiol. 2009;136(1):71–4. 10.1016/j.ijfoodmicro.2009.09.001 19775769

[23] Daayf F, Adam L, Fernando WGD. Comparative screening of bacteria for biological control of potato late blight (strain US-8), using in vitro, detached-leaves, and whole-plant testing systems. Can J Plant Pathol. 2003;3:276–84.

[24] Ma YH, Tan XY, Huang SL, Zhang X, Zang LQ, Niu XR. Identification of a biocontrol strain Z2 against pomegranate dry rot and optimization of its cultural conditions. Acta Phytopathol Sin. 2015;45(4):425–37.

[25] Xie XW, Chuan HY, Dong R, Yang J, Shi YX, Li BJ. Preparation of micropowder of Bacillus methylotrophicus WF-3 and its control effect on cucumber anthracnose. Chin J Biol Control. 2018;34(5):722–8.

[26] Adesemoye AO, Torbert HA, Kloepper JW. Enhanced plant nutrient use efficiency with PGPR and AMF in an integrated nutrient management system. Can J Microbiol. 2008;54(10):876–86. 10.1139/w08-081 18923557

[27] Saha A, Mandal P, Dasgupta S, Saha D. Influence of culture media and environmental factors on mycelial growth and sporulation of Lasiodiplodia theobromae (Pat.) Griffon and Maubl. J Environ Biol. 2008;29(3):407–10. 18972700

[28] Casals C, Elmer PAG, Viñas I, Teixidó N, Sisquella M, Usall J. The combination of curing with either chitosan or bacillus subtilis cpa-8 to control brown rot infections caused by Monilinia fructicola. Postharvest Biol Technol. 2012;64(1):126–32.

[29] Whipps JM. Microbial interactions and biocontrol in the rhizosphere. J Exp Bot. 2001;52:487–511. 10.1093/jexbot/52.suppl_1.487 11326055

[30] Samain E, Van Tuinen D, Selim S. The plant-growth-promoting rhizobacterium Paenibacillus sp. strain B2 stimulates wheat defense mechanisms against septoria leaf blotch and root colonization by Curtobacterium plantarum. Biol Control. 2017;114:87–96.

[31] Zhang H, Li G, Qin F, Zhou M, Qin P, Pan M. Castor bean growth and rhizosphere soil property response to different proportions of arbuscular mycorrhizal and phosphate-solubilizing fungi. Ecol Res. 2014;29(2):181–90.

[32] Gong AD, Li HP, Shen L, Zhang JB, Wu AB, He WJ, et al. The shewanella algae strain YM8 produces volatiles with strong inhibition activity against Aspergillus pathogens and aflatoxins. Front Microbiol. 2015;6:1091. 10.3389/fmicb.2015.01091 PMC4594021 26500631

[33] Purkayastha GD, Mangar P, Saha A, Saha D. Evaluation of the biocontrol efficacy of a Serratia marcescens strain indigenous to tea rhizosphere for the management of root rot disease in tea. PLoS One. 2018;13(2):e0191761. 10.1371/journal.pone.0191761 PMC5821441 29466418

